# Supplementary material for: Bayesian transfer in a complex spatial localization task
Source: J Vis. 2020 Jun 24;20(6):17. doi: 10.1167/jov.20.6.17 (PMC7416888; doi:10.1167/jov.20.6.17)
Supplement: Supplement 1 [file jovi-20-6-17_s001.pdf]

Supplementary material for

Title: Bayesian transfer in a complex spatial localisation task

Authors: Reneta K. Kiryakova, Stacey Aston, Ulrik R. Beierholm, Marko Nardini

Table of contents:

Appendix A: Instructions for Experiment 1 and Experiment 2

Appendix B: Control experiment (likelihood-only task)

Appendix C: Additional analyses for Experiment 1

Appendix A

Instructions in Experiment 1:

“We will ask you to play an “octopus” game!

Imagine that you are on a boat and there are 2 octopuses you are trying to find: one is white, and the other one is black. The white octopus has square tentacles and the black octopus has circular tentacles. The 2 octopuses live in different parts of the sea. Sometimes the octopuses will show their tentacles and at other times they will hide at the bottom of the sea.

Your job is to try and figure out where the octopus is!

Once you decide on a location, you can click on the green square (your fishing net), at which point you will see a red dot, which shows you the true location of the octopus on that trial. If the red dot is inside the net, then you correctly guessed the location of the octopus and you get a point!”

Instructions in Experiment 2:

“We will ask you to play an ”octopus” game!

Imagine that you are on a boat and there are 2 octopuses you are trying to find: one is white, and the other one is black. The white octopus has square tentacles and the black octopus has circular tentacles. The 2 octopuses live in different parts of the sea. Sometimes the octopuses will show their tentacles and at other times they will hide at the bottom of the sea.

[It is important to remember that one of the octopuses tends to stay in a particular area, whereas the other one moves quite a bit!]

Your job is to try and figure out where the octopus is!

Once you decide on a location, you can click on the green square (your fishing net), at which point you will see a red dot, which shows you the true location of the octopus on that trial. If the red dot is inside the net, then you correctly guessed the location of the octopus and you get a point!”

Appendix B

Control Experiment: Likelihood-only task

Participants consistently performed sub-optimally across *all* of our experiments. However, when we calculated the optimal weight on the likelihood, we did so under the assumption that people know the true values of the reliability of the sensory cue (i.e., the likelihood). As Sato and Kording (2014) point out, this is clearly not always the case: in fact, in order to perform optimally on our tasks, observers may need to learn about their likelihood variability, as well as prior variability. We, therefore, separately assessed any sensory noise that participants may have had in judging the centroid of the set of dots. If we find that subjects' estimates of the reliability of the likelihood differ from the true values, this would mean that subjects were using incorrect parameters for the task, which may have led to suboptimal performance. We then recomputed the optimal weights based on errors in observers' estimates of centroid location; we could, therefore, test whether subjects were, in fact, near-optimal, when their own sensory variability was taken into account.

Methods

Subjects (*N* = 26; 6 had participated in Experiment 2, 6 had participated in Experiment 3, and the rest had not completed any of the above tasks) were instructed to estimate the centroid of eight dots for different likelihood widths. True locations were drawn from a uniform distribution across the screen (no prior). There were 90 trials overall, with 30 trials of each likelihood width interleaved in a random order. No feedback was given.

For each participant, their error on each trial was calculated by taking the difference between the response and true location for that trial (error = response – true). Their variable error for each likelihood condition was calculated as the standard deviation of the errors. Outliers were excluded prior to calculating the variable error in the same way as described previously.

Results and Discussion of Control Experiment

Participants were significantly worse than ideal (variable error was greater than the true standard deviation of the likelihoods) in the low ($t(25) = 7.45, p < .001$)

and medium ($t(25) = 3.80, p < .001$), but not the high ($t(25) = 1.48, p = .151$) variance likelihood conditions (Figure 8).

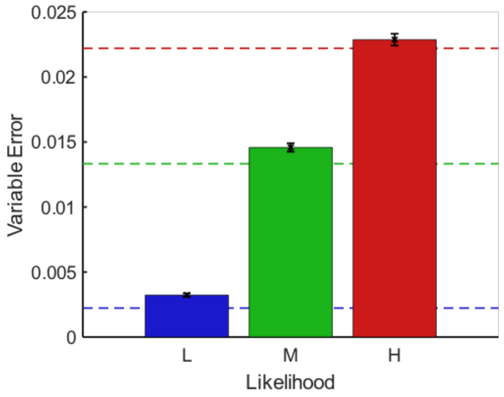

Figure 8. Variable error for each likelihood condition in the likelihood-only task. The dashed lines show the true standard deviations of the likelihood in each case (ceiling performance).

This suggests that our optimal predictions place too much weight on the likelihood, as they were calculated based only on the external variability of the sensory cue and failed to also incorporate the added variability from observer's inability to perfectly calculate the dot centroids. We, therefore, recomputed the ideal weight for the likelihood, this time using the measured likelihood variances in the control experiment; we reasoned that this calculation would give us an optimal prediction that better matches our subjects' performance. Our estimates of the likelihood variance increased by 16.66% for the low, 2.96% for the medium and 5.26% for the high likelihood. With such large differences between the true and estimated likelihood variances, we expected that the re-calculated optimal predictions (based on subjects' estimates) will be closer to the observers' data, compared to those based on the true likelihood parameters. We compared these optimal values to subjects' weights in the final block (5) in Experiment 1, and found that they were still significantly different from the empirical data when the variance of the likelihood was high or medium, irrespective of prior variance (all $p < .001$) (see Figure 9). No significant differences were observed when the prior variance was wide and the likelihood variance was low ($p = .765$).

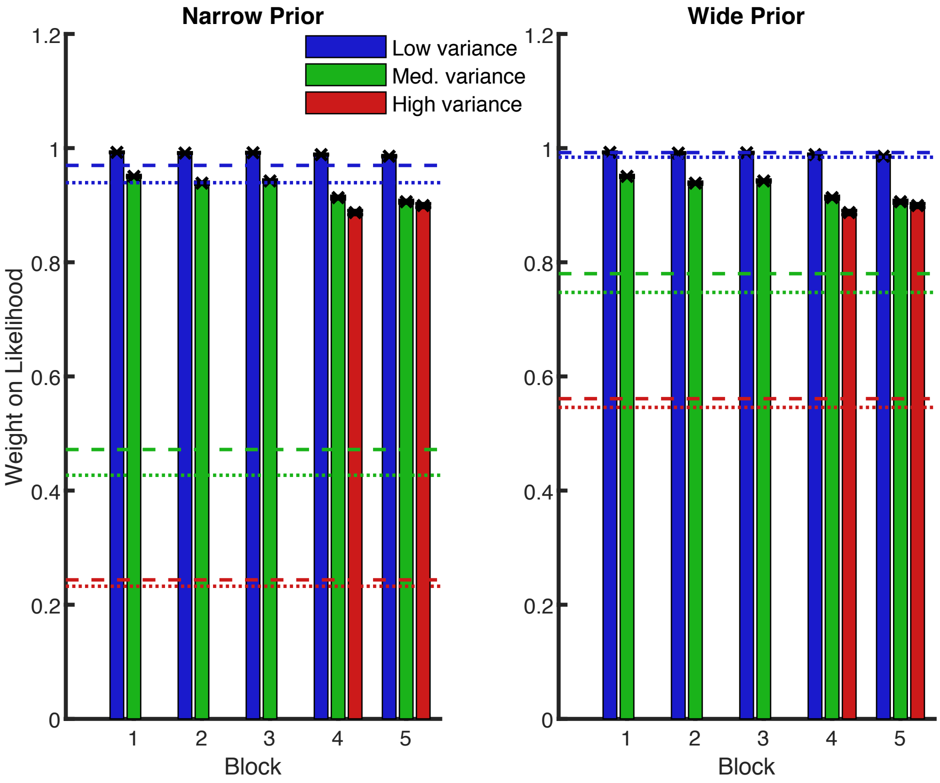

Figure 9. Mean weight placed on the likelihood information in each block of Experiment 1. Blue is low variance likelihood, green is medium variance likelihood, red is high variance likelihood. Dashed lines show optimal values. Dotted lines show optimal values, computed using measured likelihood variances in the control experiment. Error bars are +/- 1 SEM.

This pattern of results was surprisingly similar to the one we found when using the predictions of the optimal Bayesian observer, so this analysis did not affect our conclusions on the observers' suboptimal behaviour. In particular, sensory noise in determining the centroid of the "likelihood dots" does not play a major role in explaining subjects' sub-optimality in the task.

Appendix C

The observed lack of statistically significant difference in cue weights does not necessarily imply a lack of substantial difference in terms of performance (points), as previous studies have shown that participants can be "optimally lazy" by deviating from optimal performance in a way that has minimal impact on overall expected score in a task (Acerbi et al., 2017). First off, we computed the optimal response variability σ_b^2 in using both the cue (overall likelihood variability σ_o^2) and the prior as

$$\sigma_b^2 = \frac{\sigma_o^2 \, \sigma_p^2}{\sigma_o^2 + \sigma_p^2}$$

Since we are interested in the performance of the model in terms of reward, we then calculated expected gains by first computing the probability of catching an octopus on a given trial as

$$p = P\left(-\frac{w}{2} \leq X \leq \frac{w}{2}\right)$$

where p is the probability that a random draw X from a Gaussian distribution with mean μ (fixed at zero) and standard deviation σ_b^2 will fall within the "hit" distance from the true location, and that distance is half the width of the net $\frac{w}{2}$. The probability of catching the octopus p is then multiplied by the number of trials (per trial type in a block) to calculate the expected number of points.

We then compared expected reward to the average reward earned by those participants who took part in the control experiment and either Experiment 2 or Experiment 3 (*N* = 12; in block 5 only), and found that optimal integration of the sensory cue and prior knowledge (according to participants' overall noise in using the cue) resulted in an expected reward that was higher than what our participants achieved, but only when the variance of the prior was narrow; when the prior variance was wide, they matched quite well; see Figure 10. This result is particularly challenging for the notion that people may be "optimally lazy", as this case would result in predicted and obtained reward values being equal. It can be seen that contrary to these predictions, our observers were clearly worse than the optimal observer, and could earn more points when the variance of the prior was narrow; it is, therefore, unlikely that their suboptimal performance could be explained by them being "optimally lazy" (Acerbi et al., 2017).

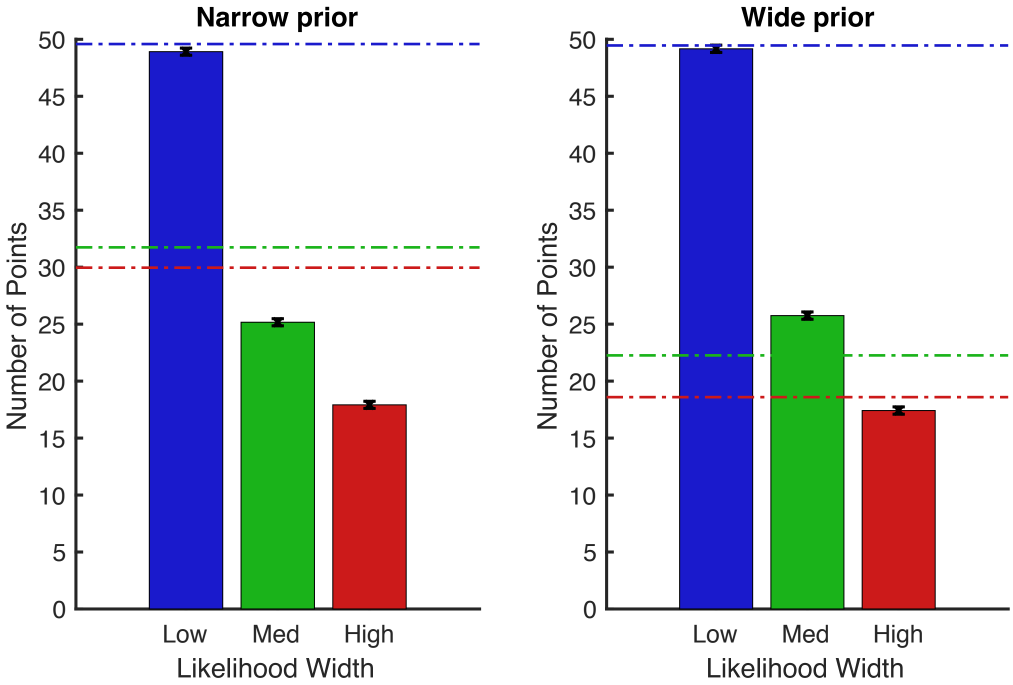

Figure 10. Mean number of points earned in Block 5 for participants who took part in Experiment 2 or Experiment 3 and the control task (*N* = 12). Blue is low variance likelihood, green is medium variance likelihood, red is high variance likelihood. Dot-dashed lines show optimal reward values, taking into account participants' overall noise. Error bars are +/- 1 SEM.
